# Individualized treatment with transcranial direct current stimulation in patients with chronic non-fluent aphasia due to stroke

**DOI:** 10.3389/fnhum.2015.00201

**Published:** 2015-04-21

**Authors:** Priyanka P. Shah-Basak, Catherine Norise, Gabriella Garcia, Jose Torres, Olufunsho Faseyitan, Roy H. Hamilton

**Affiliations:** ^1^Neurology, University of PennsylvaniaPhiladelphia, PA, USA; ^2^Perelman School of MedicinePhiladelphia, PA, USA; ^3^Temple University School of MedicinePhiladelphia, PA, USA; ^4^Neurology, NYU Langone Medical CenterNew York, NY, USA; ^5^Physical Medicine and Rehabilitation, University of PennsylvaniaPhiladelphia, PA, USA

**Keywords:** tDCS, aphasia, stroke, language disorders, neurorehabilitation

## Abstract

While evidence suggests that transcranial direct current stimulation (tDCS) may facilitate language recovery in chronic post-stroke aphasia, individual variability in patient response to different patterns of stimulation remains largely unexplored. We sought to characterize this variability among chronic aphasic individuals, and to explore whether repeated stimulation with an individualized optimal montage could lead to persistent reduction of aphasia severity. In a two-phase study, we first stimulated patients with four active montages (left hemispheric anode or cathode; right hemispheric anode or cathode) and one sham montage (Phase 1). We examined changes in picture naming ability to address (1) variability in response to different montages among our patients, and (2) whether individual patients responded optimally to at least one montage. During Phase 2, subjects who responded in Phase 1 were randomized to receive either real-tDCS or to receive sham stimulation (10 days); patients who were randomized to receive sham stimulation first were then crossed over to receive real-tDCS (10 days). In both phases, 2 mA tDCS was administered for 20 min per real-tDCS sessions and patients performed a picture naming task during stimulation. Patients' language ability was re-tested after 2-weeks and 2-months following real and sham tDCS in Phase 2. In Phase 1, despite considerable individual variability, the greatest average improvement was observed after left-cathodal stimulation. Seven out of 12 subjects responded optimally to at least one montage as demonstrated by transient improvement in picture-naming. In Phase 2, aphasia severity improved at 2-weeks and 2-months following real-tDCS but not sham. Despite individual variability with respect to optimal tDCS approach, certain montages result in consistent transient improvement in persons with chronic post-stroke aphasia. This preliminary study supports the notion that individualized tDCS treatment may enhance aphasia recovery in a persistent manner.

## Introduction

Aphasia is a common and often debilitating consequence of stroke, typically arising from damage to perisylvian structures in the left hemisphere. Currently, the most widely employed treatment for aphasia following stroke is speech and language therapy (SLT), however, the magnitude (Basso, 2010) and duration (Marangolo et al., 2013b) of improvement attributable to these therapies is variable and can be fairly limited. In the last decade, a growing body of evidence has emerged that suggests that non-invasive brain stimulation can be used to enhance recovery from stroke-induced deficits such as neglect (Koch et al., 2012; Mylius et al., 2012; Sunwoo et al., 2013), paresis (Mansur et al., 2005; Ayache et al., 2012; Khedr et al., 2013), and aphasia (Naeser et al., 2005; Baker et al., 2010; Medina et al., 2012). With respect to aphasia treatment, transcranial direct current stimulation (tDCS) is an especially attractive brain stimulation approach, owing to its safety, portability, low cost, and the ease with which it can be paired with existing interventions such as speech and language therapy.

During tDCS administration, a small electrical current is passed through brain structures via electrodes placed on the scalp. This current is insufficient to result in direct neuronal depolarization; rather it is thought to induce incremental shifts in the resting membrane potentials of large numbers of neurons under the electrodes. These modest shifts in the resting state are sufficient to drive measurable changes in motor neurophysiology and cognitive functions (Nitsche and Paulus, 2000; Feng et al., 2013; Pellicciari et al., 2013). The effects of tDCS have been observed up to an hour following a single stimulation session and may persist for days or even months after multiple days of stimulation (Reis et al., 2009). It is believed that the polarity of the electrodes determine their effects on cortical activity. Anodal-stimulation has been associated with facilitative effects on cortical activity (Cuypers et al., 2013), while cathodal-stimulation has been associated with inhibitory effects (Chrysikou et al., 2013). However, recent findings call into question this straightforward relationship. Factors such as current intensity, stimulation duration, and training tasks—typically performed during stimulation—can impact the excitatory or inhibitory aftereffects of stimulation in unpredictable ways, especially with respect to cathodal tDCS (Jacobson et al., 2012; Batsikadze et al., 2013; de Aguiar et al., 2015).

Promising results for tDCS treatment have been reported for patients with subacute (You et al., 2011), and chronic (Marangolo et al., 2013a) post-stroke aphasia, and for those with non-fluent (Baker et al., 2010) and fluent (Volpato et al., 2013) aphasia syndromes. Importantly, there is considerable variability in the approaches adopted by different investigators with respect to the cortical hemisphere stimulated, the polarity of stimulation, and the presumed stimulation targets. A number of these studies have been motivated by an interhemispheric inhibition model of language recovery, in which either enhancement of the left frontal or temporal activity or suppression of the maladaptive right-hemisphere hyperactivity may result in more robust compensatory recovery of left-hemisphere perilesional regions (Chrysikou and Hamilton, 2011). Consequently, these studies have employed either left-anodal or right-cathodal montages (Monti et al., 2013). One notable exception is Monti et al. (2008), who found that cathodal tDCS applied over the lesioned left frontotemporal area lead to a significant increase in correct responses during a picture-naming task when compared to sham and anodal tDCS conditions.

While the interhemispheric inhibition model has been highly influential with respect to treatment studies of aphasia, converging evidence from functional neuroimaging experiments, behavioral studies, and transcranial magnetic stimulation (TMS) investigations suggests that multiple mechanisms of neuroplasticity underlie aphasia recovery. Data consistently supports the compensatory role of the left-hemisphere perilesional areas (Heiss and Thiel, 2006); however, the contribution of the right-hemisphere in aphasia recovery appears to be more varied and complex (Schlaug et al., 2011; Torres et al., 2013). In addition to the interhemispheric inhibition model described above, another theory postulates that the right hemisphere plays a compensatory role in reorganized language networks (Hartwigsen et al., 2013). Still others have argued that increased right hemisphere activity contributes beneficially to post-aphasia recovery, but that inefficiencies in remodeled language networks may impose limits on that recovery (Turkeltaub et al., 2011). These models of language plasticity are not mutually exclusive, and in fact evidence suggests multiple stroke recovery mechanisms can be engaged within the same individual (Turkeltaub et al., 2012). This suggests that patients may respond differentially to different tDCS approaches but this possibility has not been systematically investigated, and represents a limitation in the non-invasive brain stimulation literature on aphasia recovery.

Another area that remains to be fully explored is the long-term efficacy of tDCS in ameliorating aphasia symptom severity in a group of patients. While many investigators have reported positive changes in language measures either immediately after receiving tDCS (Fiori et al., 2011) or within 1 or 2 weeks of stimulation (Baker et al., 2010), only a few recent studies have explored the potential benefits of tDCS over longer periods of time (Marangolo et al., 2013a,b; Polanowska et al., 2013; Manenti et al., 2015).

The current study sought to address these two important gaps in a two-phase investigation. In Phase 1, subjects underwent anodal- and cathodal-tDCS of the left and right prefrontal areas, as well as a sham condition, in separate sessions. A preferred electrode montage was established for each subject by assessing transient improvement on a picture-naming task. In Phase 2—a randomized and sham-controlled phase—tDCS was administered over 10-days using the optimal electrode configuration that was identified in Phase 1. Subjects were reassessed 2-weeks and 2-months post-stimulation, after which patients in the sham arm crossed over into the real arm of the study. Based on our central hypothesis that aphasic patients employ different recovery mechanisms to varying degrees (Turkeltaub et al., 2012; Torres et al., 2013), we predicted heterogeneity across patients in their response to different tDCS montages in Phase 1. In light of prior investigations of tDCS as a potential treatment for aphasia, we also predicted that repeated stimulation with each individual patient's optimal montage would lead to persistent improvement in aphasia severity in Phase 2.

## Materials and methods

### Subjects

**Figure 1:**
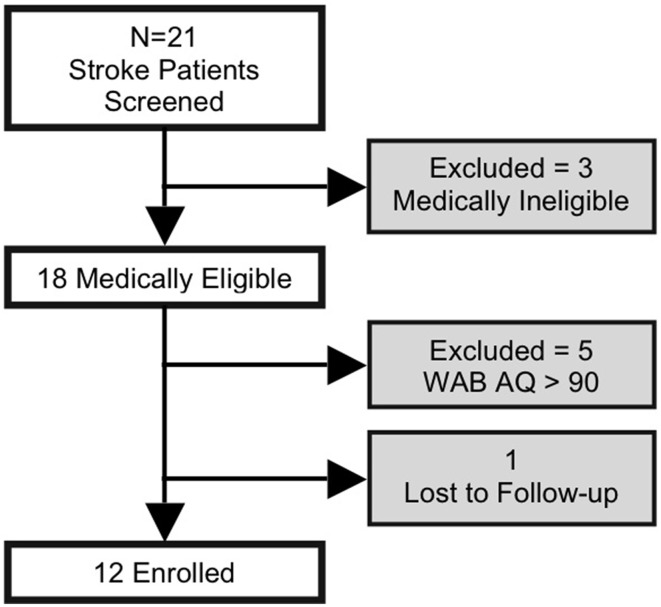
**Flow chart indicating the number of study subjects who were screened and enrolled; subjects in shaded boxes were excluded from analyses**.

Subjects had a history of a single left-hemispheric chronic stroke (≥6 months post stroke-onset), had mild-to-severe non-fluent aphasia, were premorbidly right-handed (Edinburgh Handedness Inventory) (Oldfield, 1971), and had no concurrent history of neurological, psychiatric or unstable medical conditions, or any contraindication to either MRI or tDCS. Aphasia symptoms and severity were screened using the Western Aphasia Battery (WAB) (Kertesz, 1982), to avoid ceiling effects, individuals with a WAB-Aphasia Quotient (WAB-AQ) above 90 were excluded. Out of 21 screened subjects, 3 were medically ineligible, 5 did not meet the eligibility criterion, and 1 was lost to follow-up, resulting in 12 enrolled subjects (2 females; age: 63.6 ± 8.6, range = 53–78 years; Figure 1). None of the enrolled subjects initiated new language therapies or engaged in other treatment studies during the course of the study. Additional demographic information and lesion descriptions for the 12 enrolled subjects are provided in Table 1 (also refer to the Supplementary Figure 1). A single neurologist (RHH) used clinical scans (MRI/CT) obtained during or after each patient's medical treatment for stroke to delineate lesion locations.

**Table 1:** **Demographics and clinical variables of enrolled study subjects**.

**Subject**	**Sex**	**Age (years)**	**Time since stroke (months)**	**Lesion distribution**	**Lesion volume (cm^3^)**	**WAB-AQ**
P1	M	65	27	^a^Left MCA	–	29.4
P2	M	73	52	Anterior MCA distribution involving posterior IFG, insula, subcortical white matter and basal ganglia Temporal and parietal cortex spared	121.79	28.4
P3	M	61	12	Large fronto-temporo-parietal lesion involving STG, parietal cortex, left IFG and subcortical white matter Deep gray structures and thalamus spared	266.29	23.2
P4	M	53	66	Fronto-parietal cortical and subcortical, including internal capsule, basal ganglia, anterior IFG	165.49	87.8
P5	M	54	7	Large fronto-temporo-parietal lesion involving STG, parietal cortex, IFG, and subcortical white matter Caudate and thalamus spared	271.02	38.9
P6	M	67	10	Fronto-parietal lesion involving supramarginal gyrus, temporo-parietal-occipital junction, insula, IFG, and underlying subcortical white matter Basal ganglia and thalamus spared	89.8	69.5
P7	M	76	101	Fronto-temporo-parietal subcortical, including corona radiata Internal capsule, deep gray structures, and IFG spared	145.94	69.6
P8	M	61	28	Fronto-parietal lesion involving sensorimotor and superior parietal cortices, and subcortical white matter IFG, inferior parietal gyrus, temporal cortex, deep gray structures, and thalamus spared	134.04	83
P9	F	63	7	Fronto-parietal lesion involving posterior STG, left parietal, sensorimotor and supplementary motor cortices Deep gray structures and IFG spared	264.86	40.7
P10	M	59	9	Large fronto-temporo-parietal lesion involving STG, parietal cortex, IFG, and subcortical white matter Deep gray structures and thalamus spared	197.18	33.4
P11	M	53	44	Frontal lobe involving IFG and middle frontal gyrus, sensorimotor cortex, subcortical white matter, and caudate	175.16	78.8
P12	F	78	9	Posterior STG and left parietal sulcus including supramarginal gyrus Deep gray structures and IFG spared	209.43	57.5
Mean (SD)		63.6 (8.6)	31.0 (29.7)		185.5 (62.3)	53.3 (23.6)

*MCA, Middle cerebral artery; IFG, Inferior frontal gyrus; STG, Superior temporal gyrus; SD, Standard deviation; P1–P7 entered Phase 2*.

a *Structural images were reviewed during subject screening and enrolment but were not available during data analysis or results reporting*.

**Figure 2:**
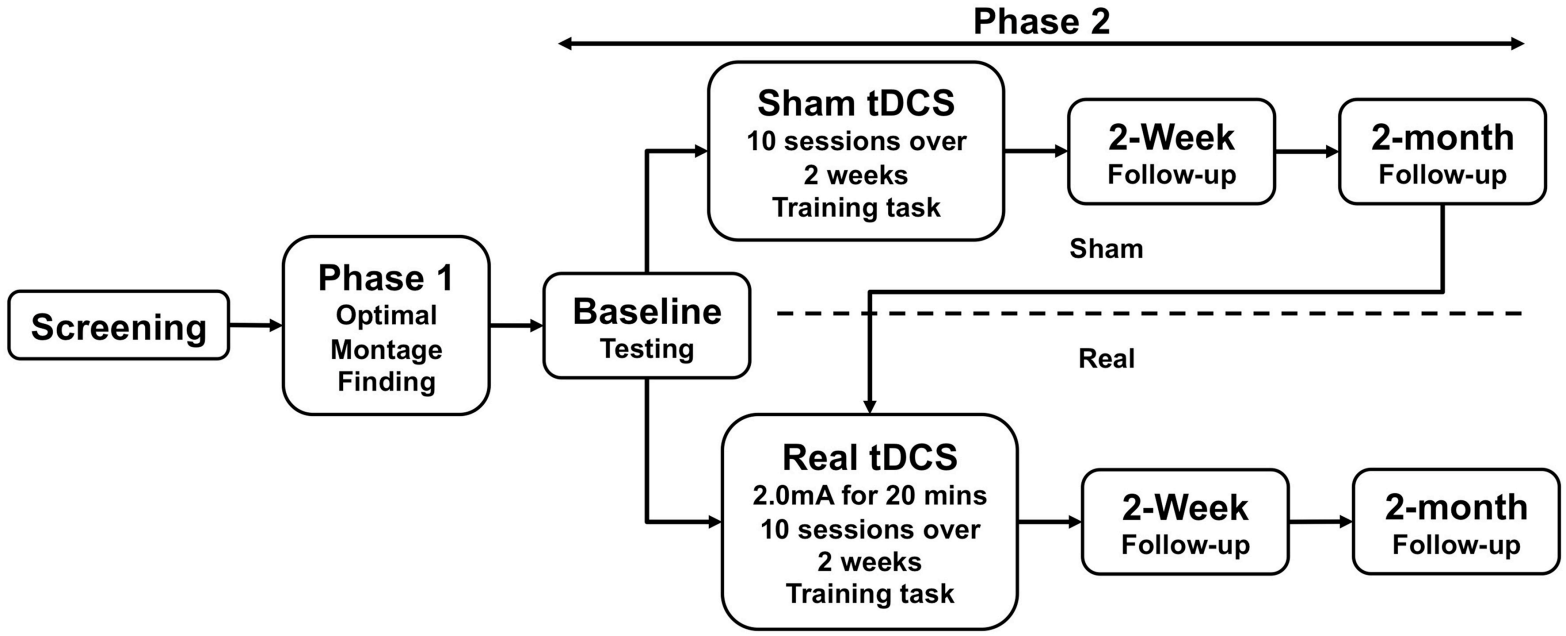
**Overview of study events**.

The study was approved by the Institutional Review Board of the University of Pennsylvania. All subjects provided informed consent. An overview of study events is illustrated in Figure 2.

### Western aphasia battery

The WAB (Kertesz, 1982) was administered during subject screening, and was also the instrument employed in Phase 2. The WAB characterizes: (1) spontaneous speech through a picture description and responses to questions regarding personal facts, (2) auditory comprehension through responses to yes/no questions, object identification, and sequential commands, (3) repetition through repeating single words, compound words and sentences, and (4) naming and word finding through object naming, word fluency, sentence completion, and responsive speech. The composite score of these four subtests forms the WAB Aphasia Quotient (WAB-AQ), which is an overall rating of aphasia severity and which served as our main outcome measure in Phase 2. In addition, to depict specific changes in language functions as a function of optimal tDCS, WAB subtest scores were also analyzed in Phase 2.

### Phase 1

#### Stimulation parameters

In five non-consecutive days, subjects underwent tDCS with four active conditions and one sham condition, using a Magstim Eldith 1 Channel DC Stimulator Plus (Magstim, Whitland, UK); these sessions were separated on average by 6.85 (± 5.9) days. In each montage, the active electrode was placed over a frontal lobe site identified using the10–20 EEG measurement system (F3 = left-frontal; F4 = right-frontal). These frontal sites overlie brain areas that are superior to the inferior frontal gyrus, which is often lesioned in patients with non-fluent aphasia. We theorized that F3 stimulation would likely be associated with perilesional stimulation in the left hemisphere. The reference electrode was placed over the contralateral mastoid. This site was chosen in order to minimize current flow in right frontal lobe during left frontal stimulation and vice versa, in order to be able to isolate stimulation to a single prefrontal cortex (Datta et al., 2011). We also hypothesized that current would flow inferiorly from the frontal lobe site to the contralateral mastoid, potentially including language-relevant targets such as the inferior frontal gyrus (cf. Datta et al., 2011; Chrysikou et al., 2013). The four active conditions were F3-anode, F3-cathode, F4-anode, and F4-cathode. In line with widely used and safe parameters (Brunoni et al., 2011; Kessler et al., 2012; Russo et al., 2013), stimulation was delivered for 20 min at 2.0 mA using 5 × 5cm^2^ sponge electrodes (current density: 0.80 μA/mm^2^) with a 30-s ramp-up and ramp-down period. For sham, stimulation was ramped up to 2.0 mA and then down to 0 mA in the first minute of stimulation, and subjects were randomized to receive either F3-anode and cathode on right mastoid or F4-anode and cathode on left mastoid. The order of five conditions was counterbalanced across subjects, who were blinded to real or sham-tDCS (Gandinga et al., 2006). The person administering tDCS was not blinded to tDCS conditions.

#### Identification of optimal montage

Picture-naming ability was assessed before and immediately after each stimulation session with an 80-item task employing images from the International Picture Naming Project database (IPNP) (Szekely et al., 2004). The 80-item picture sets were matched for word-frequency, word-length (Supplementary Table 1), and semantic category. Subjects received no feedback regarding their performance. Responses were recorded digitally and later scored offline by the investigator who was blinded to the montage (left/right anode, left/right cathode or sham) and session (pre/post). Subject's responses were scored as correct if the name they provided for the picture matched the target word provided in the IPNP database or if it was a close synonym. A failure to respond to a picture prompt was scored as “no response.” Partial responses or descriptions of pictures were scored as incorrect.

The difference between the number of items that were named correctly before and following each stimulation session was calculated (post- vs. pre-stimulation; refer to Supplementary Table 2 for more details). To examine variability in responsiveness to tDCS, we first compared the change in subjects' performance across all active montages with respect to the sham montage. Second, in line with previously reported methods (Naeser et al., 2005; Medina et al., 2012), an electrode montage was defined as optimal for each subject if the subject (1) showed the greatest change in accuracy after stimulation using a particular montage and (2) if the accuracy post-stimulation with that montage was ≥ the upper limit of the 90% confidence interval (CI) of pre-stimulation performance across all montages (mean pre-stimulation accuracy + 1.645 ^*^ standard deviation across pre-stimulation sessions); both these requirements had to be met for the montage to qualify as optimal.

#### Training task

During the 20 min of active- or sham-tDCS (for both Phase 1 and Phase 2—details below), subjects completed a picture-naming task that was based on (but was not identical to) constraint-induced language therapy (CILT), in that it minimized non-verbal communication between subjects and the experimenter (Pulvermuller et al., 2001; Maher et al., 2006). Subjects were shown 20 black-and-white images taken from the IPNP database, one at a time. A physical barrier between subjects and the experimenter was erected to constrain subjects to produce verbal responses and also to prevent unanticipated visual cues from the experimenter (Maher et al., 2006). This feature is central to CILT, whereby subjects' responses are limited to the spoken modality rather than alternative modes of communication such as gestural responses. Another important feature of CILT is ensuring that the task is sufficiently challenging. In line with this requirement, the naming items increased in difficulty over the course of stimulation. Unlike CILT however, we did not provide any feedback regarding the accuracy of subjects' responses and dual-card task was replaced with a picture naming task.

The 20-item lists used during stimulation were not repeated across the training sessions. Owing to the limited size of the IPNP corpus, the stimuli used as training items comprised a small subset of the items used during pre- and post-stimulation testing. However there were no systematic differences in number of repeated items across sessions.

### Phase 2

#### Sham-controlled crossover trial

In Phase 1, 7/12 subjects exhibited significant transient improvement in naming after stimulation with at least one active electrode arrangement. Six subjects entered Phase 2; one subject declined further study participation. Another subject completed only the sham arm of Phase 2 (described below), but declined to participate in the real-tDCS phase, leaving five who completed the study in its entirety.

Each of the six subjects who entered Phase 2 was randomized to receive either real-tDCS treatment (*n* = 3), or sham stimulation followed by real-tDCS (*n* = 3). To establish a stable pre-tDCS baseline of aphasia severity, the WAB was administered 3 times in separate behavioral sessions prior to initiating real or sham treatment; the average interval between initial and final baseline testing sessions was 7 days (±4.6 days). During treatment, subjects received tDCS for a total of 10 days (Monday–Friday for two consecutive weeks). Stimulation parameters were identical to Phase 1. Subjects engaged in the training task described above (same as Phase 1) during both the real- and sham-tDCS sessions (Maher et al., 2006). Subjects repeated assessment with the WAB, 2 weeks and 2 months after treatment. Following 2-month follow-up, subjects in the sham arm crossed over into the real arm and received real-tDCS, followed by 2-week and 2-month follow-up assessments (Figure 2). Subjects who initially received real-tDCS were blinded to their treatment condition. Subjects receiving sham stimulation were blinded to their condition until they crossed over into the real arm of the study, at which point they were by necessity informed of their condition (as required by our IRB). Change in WAB-AQ was the principle outcome measure for this phase.

## Results

The R Development Core Team (2013) was employed for all statistical analyses.

### Phase 1

**Figure 3:**
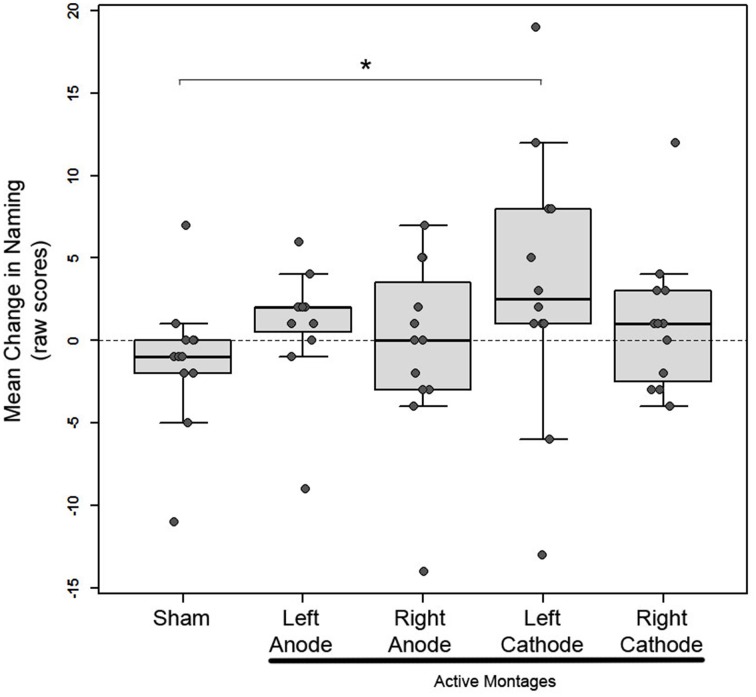
**Phase 1: Mean change in picture-naming in 12 subjects after stimulation with 1 sham and 4 active montages in box plots; box height represents the interquartile range, the black line within the box represents the median, the whiskers represent the upper and lower ranges**. Each patients' mean change is superimposed on the box plots as solid gray circles. Asterisk indicates statistical significance (^*^p < 0.05) between the sham and left-cathodal montages.

To examine heterogeneity in responsiveness to tDCS with different montages, we implemented mixed linear effects (MLE) analysis in 12 subjects. We chose the MLE over the traditional repeated measures ANOVA (rmANOVA) because the former is advantageous in two important ways: (1) it is a more powerful statistical approach for detecting within- as well as between-subject differences in groups with small and unbalanced sizes, and (2) it takes into account subject-level or individual variability (random intercept) to reliably depict differences across different conditions (Goedert et al., 2013). In a random-intercept model, we included the fixed effects of montages (sham, left-anode, left-cathode, right-anode, right-cathode), order of study visits and two clinical variables—lesion volume and time since stroke, and the random effects of subjects; change in raw scores of naming (post- vs. pre-tDCS) was included as the dependent variable. The model indicated that the change in naming was greater after stimulation with the left-cathode montage as compared to the sham montage, whereby the model estimate was positive and statistically significant (*b* = 5.14, *SE* = 2.29, *p* = 0.029; Figure 3). Notably, out of the 12 subjects, 10 exhibited a positive change in naming after stimulation with the left-cathode montage while only two subjects responded to it negatively (Figure 3). In these 10 patients, the positive change did not necessarily reflect the greatest change compared to other montages and therefore left-cathode montage did not always qualify as the optimal montage (see below). Furthermore, the effects of clinical variables (lesion volume and time since stroke) and order were not significant (*p* > 0.05). Since our primary interest was in personalizing treatment with tDCS in Phase 2, we next determined the montage that each subject responded to optimally.

As reported earlier, 7/12 subjects in Phase 1 responded to at least one active stimulation montage optimally, while three did not to any montages, and one subject responded to sham. One other subject (P10) was excluded from further analysis because pre-stimulation he could not name any pictures (naming accuracy = 0), and post-stimulation his performance did not substantially improve across any of the montages. In line with our predictions, the optimal montage varied considerably across the seven subjects who responded: three subjects responded optimally to left-anode (P1, 2, 4), three subjects to left-cathode (P 5, 6, 7), and one subject to right-cathode (P3; refer to Supplementary Figure 1 for electrode locations). Only those subjects who responded to at least one montage entered Phase 2 and were stimulated using their optimal montage, while subjects who did not respond to any montages were not included in Phase 2.

**Figure 4:**
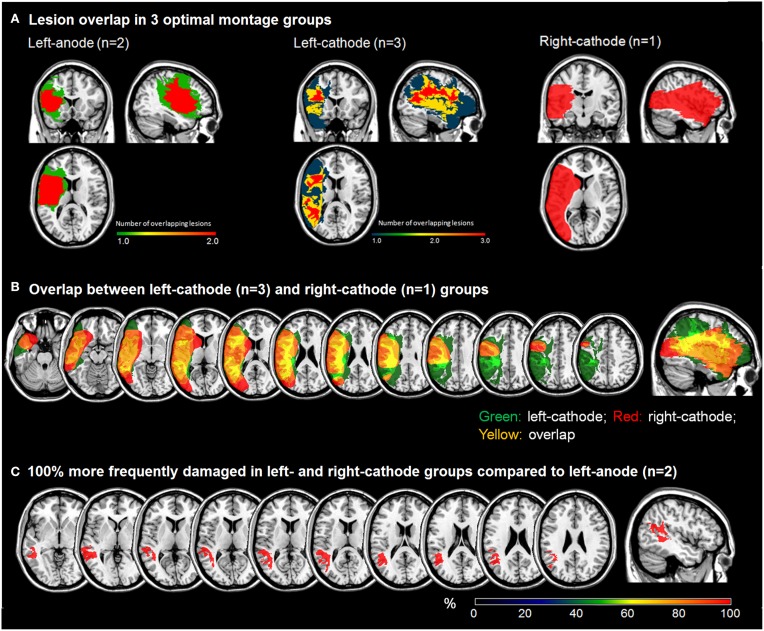
**Phase 1: (A) Lesion overlap plots for different optimal montage-groups; (B) Lesion overlap between the cathodal montage groups; (C) Subtraction plot comparing left-anodal group to cathodal montage group**.

As exploratory analyses, we examined the maximal lesion overlap and subtraction in three montage-groups in patients who responded to at least one montage optimally. Visual inspection of lesion overlap revealed that lesions in left-anode group (*n* = 2) were confined within the frontal areas, whereas left- (*n* = 3) and right-cathode (*n* = 1) groups presented with large lesions encompassing several areas along the frontotemporal network (Figure 4A). In the left-cathode group, lesions extended more superiorly and medially including parietal along with the frontotemporal areas (Figure 4B). To qualitatively compare lesion overlaps, subtraction analysis between left-anode and cathodal stimulation groups, irrespective of the hemisphere of stimulation, was conducted. This analysis revealed that superior and middle temporal areas were more frequently damaged in patients who responded to cathodal stimulation in general (Figure 4C). Overall, this preliminary evidence is consistent with prior studies suggesting that lesion location and size critically impact mechanisms of neuroplasticity in language recovery (Heiss and Thiel, 2006), and that these factors in turn can influence how patients respond to different tDCS approaches (Anglade et al., 2014).

### Phase 2

Test-retest reliability, estimated using Pearson's correlation coefficient, was 0.958 (*n* = 6) indicating a high-level of stability of WAB across repeated administrations over 3.75 months (±1.8); we compared WAB-AQ obtained at screening and the third baseline. Moreover, mean WAB-AQ did not differ across the three baseline sessions [*F*_(2, 10)_ = 0.696, *p* = 0.521; refer to Supplementary Table 3 for more details], suggesting stable language performance prior to initiating treatment with tDCS.

To evaluate treatment effects with real- vs. sham-tDCS, we compared two groups: those who received only real-tDCS (*n* = 3) and those who received sham-tDCS before crossing over to real-tDCS (*n* = 3), using MLE analysis. In a random intercept model, we included the fixed effects of testing sessions (baseline, 2-week, 2-month), stimulation group (real, sham) and session by group interaction, and the random effects of subjects (Goedert et al., 2013); mean of three baseline assessments was used for this analysis. A session by group interaction was significant (Table 2; *b* = 2.32, *SE* = 0.89, *p* = 0.023) indicating that changes in WAB-AQ between the real and sham groups were different across the testing sessions. *Post-hoc* Tukey's contrasts showed that compared to the baseline (*M* = 52.83 ± 29.0), there was a trend toward improvement at 2-weeks (*M* = 58.97 ± 26.7) and a significant improvement at 2-months (*M* = 62.83 ± 24.1) in the real-tDCS group. Mean WAB-AQ scores did not differ across 2-week (*M* = 63.03 ± 33.3) or 2-month (*M* = 59.1 ± 30.8) sessions in the sham group compared to the baseline (*M* = 60.03 ± 28.3).

**Table 2:** **Summary of the fixed and random effects in the mixed linear effects model in Phase 2**.

**Dependent variable: WAB AQ**				
**Fixed Effects**	**Coefficient (*b*)**	***SE***	***t***	***Pr*(>|*t*|)**
Intercept	59.65	9.65	6.18	<0.001
Session	1.57	0.88	1.78	0.10
Group	−1.27	9.69	−0.13	0.90
Session^*^Group	2.32	0.89	2.61	0.023
**Random Effects**	**Variance**			
Subject intercept	543.67			
Residual	13.11			

Session has three levels (baseline, 2-week and 2-month), and stimulation group has two levels (real and sham). See text for details.

* *Signifies an interaction*.

We subsequently examined changes in WAB-AQ scores in all subjects in Phase 2 (*n* = 5) who underwent real tDCS, irrespective of whether it was preceded by sham. Because subjects who received real stimulation after sham differed from those receiving only real-tDCS both in number of exposures to the WAB and blinding status (see Methods above), we first compared the mean baseline and relative change of WAB-AQ from baseline to 2-weeks and baseline to 2-months by two-sided *t*-tests after receiving real-tDCS; relative change was computed by taking the difference between the WAB-AQ scores obtained at 2 week (or 2 months) and mean of baseline scores and dividing it by the mean of baseline scores. These comparisons were non-significant (all *p* > 0.05), which reduces the possibility that the observed improvement could be confounded by practice effects because of extra exposures to WAB in some patients.

**Figure 5:**
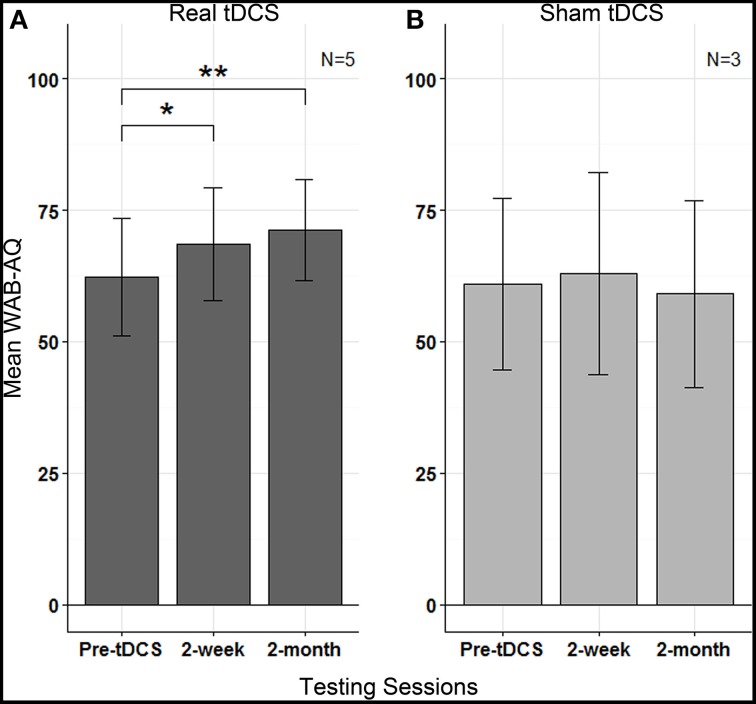
Phase 2: Mean WAB-AQ scores in (A) real tDCS and (B) sham tDCS groups at pre-tDCS, 2 weeks and 2-months; vertical lines represent standard errors, and asterisks indicate statistical significance (^**^p < 0.001, ^*^p < 0.05).

To further control for the difference in WAB exposures between groups and for practice effects, we conducted a random-intercept analysis (*n* = 5) in which the most recent WAB performance preceding real stimulation was used for comparison. Thus, for subjects who underwent real-tDCS initially, WAB-AQ obtained at the third baseline was used for comparison with post-treatment sessions, while for subjects who underwent sham prior to real-tDCS, WAB-AQ obtained 2-months after termination of sham-tDCS was used. This analysis revealed a significant effect of testing sessions (*b* = 3.73, *SE* = 0.99, *p* = 0.003) for the real-tDCS group (Figure 5A). Tukey's contrasts revealed that mean WAB-AQ scores differed between baseline and 2-weeks (*b* = 6.26; *z* = 2.63, *p* = 0.023) and baseline and 2-months (*b* = 8.88, *z* = 3.73, *p* < 0.001). This finding suggests that there was a statistically significant amelioration in aphasia severity after receiving real-tDCS in these patients. Similar analysis for WAB subtests in the real-tDCS group revealed a significant effect of sessions for spontaneous speech (*b* = 1.18, SE = 0.34, *p* = 0.0194), and a trend for naming (*b* = 0.40, *SE* = 0.15, *p* = 0.084) and repetition (*b* = 0.17, SE = 0.06, *p* = 0.076) subtests, while auditory comprehension did not change (*b* = 0.12, *SE* = 0.08, *p* = 0.46). The change in these subtests reveal improvements in language submeasures, especially those related to speech content and fluency, that impacted the overall amelioration in aphasia severity in our patients after receiving real-tDCS.

In a separate analysis for the sham group, the effect of sessions was not significant ( *b* = −0.78, *SE* = 1.28; *p* = 0.463), revealing no change in WAB-AQ (Figure 5B). None of the WAB subtest scores after sham stimulation were significant: spontaneous speech (*b* = 0, *SE* = 0.52, *p* = 0.26), naming (*b* = −0.17, *SE* = 0.09, *p* = 0.37), repetition (*b* = −0.18, *SE* =0.18, *p* = 0.64) and auditory comprehension (*b* = −0.035, *SE* = 0.12, *p* = 0.45).

## Discussion

Our results extend prior work exploring the use of tDCS as a therapy for chronic aphasia in three critical ways. First, we observed variability in how subjects respond to different electrode arrangements for administering tDCS, as judged by transient improvement in picture-naming immediately following stimulation. This demonstrates that individuals with chronic non-fluent aphasia differ with respect to their response to specific tDCS montages, which may reflect differences in neural mechanisms of aphasia recovery (Torres et al., 2013). Second, the most consistent montage for eliciting transient improvement was cathodal-stimulation of the left-frontal lobe, suggesting a common pattern of performance across most subjects despite individual variability. Third, by following patients for at least two months after the end of stimulation, our study adds to a small but growing literature suggesting that tDCS may facilitate longer-lasting changes in language ability.

Our finding that, on average, naming improvement was most pronounced after left-frontal cathodal-stimulation compared to sham stimulation was somewhat unexpected. This finding does not reconcile easily with an aphasia recovery interhemispheric inhibition model, which predicts two main therapeutic approaches: facilitation of compensatory left-hemisphere activity or suppression of deleterious right-hemisphere activity. However, very few investigators have put this model directly to the test by administering cathodal-stimulation to the left-hemisphere in aphasic patients. A study by Monti et al. (2008) stands out in this regard. Consistent with our results, they also found that subjects showed an immediate benefit in picture-naming specifically after cathodal-stimulation. To our knowledge, no other studies have systematically compared left- and right-sided cathodal- and anodal-stimulation. Importantly, our results suggest that the interhemispheric inhibition model that has motivated brain stimulation studies in aphasia for over a decade is not wholly adequate for predicting which stimulation approaches are likely to be most effective in individuals with aphasia.

Several potential hypotheses could account for Phase 1 findings. First, subthreshold inhibitory modulation of left-hemisphere cortical circuits may improve language performance by suppressing spurious neural activity in damaged and reorganized language networks, in effect increasing the signal-to-noise ratio of task-relevant neural activity (Moos et al., 2012; de Aguiar et al., 2015). Second, Monti et al. (2008) put forth a somewhat different, but not mutually exclusive explanation, speculating that left cathodal-stimulation may have an inhibitory effect on inhibitory intrahemispheric intracortical connections (Monti et al., 2008), resulting in increased overall activation of language-related left perilesional areas. Third, suppression of perilesional activity may facilitate activity in intact right-hemisphere language homologs by releasing them from left-to-right transcallosal inhibition (Schlaug et al., 2011; Turkeltaub et al., 2011, 2012). However, this explanation is weakened by the fact that direct anodal-stimulation of the right-hemisphere failed to elicit a measurable improvement. Notably, the second-best response in patients who responded to left-cathode was invariably with the right-anode montage (3/3). Fourth, cathodal-stimulation delivered at higher intensities and for longer durations (2.0 mA and 20 min—parameters in this study and Monti et al., 2008) can potentially have excitatory rather than inhibitory effects on cortical neurophysiology (Batsikadze et al., 2013). Thus, it is conceivable that the transient naming improvement after left cathodal-stimulation in our cohort was actually driven by excitation of perilesional areas. This account, however, is also weakened by the fact that the patients who responded to left-cathode did not respond measurably to direct facilitation with the left-anode montage. Lastly, the polarity-dependent tDCS effects observed in motor studies are generally less consistent in studies of cognition, especially the putative inhibitory effects of cathodal-stimulation, a notion that is supported by recent meta-analysis of tDCS studies (Jacobson et al., 2012).

We observed variability in optimal response to different montages, which may be linked to differences in lesion location and distribution across patients. Our preliminary findings suggested that patients exhibiting differential patterns of damage in the frontotemporal language areas may respond to tDCS differently. In accordance with the hierarchical model of recovery (Rosen et al., 2000; Heiss and Thiel, 2006), which predicts that recovery after perturbation in a small region may rely on the recruitment of residual language and perilesional areas, in our patients we observed that those who responded to left-anodal or facilitation of left frontal areas exhibited small lesions confined within the frontal areas. Conceivably, left anodal stimulation in these patients facilitated recruitment of spared areas, specifically the temporal language networks, resulting in the observed improvement. In contrast, lesion patterns in patients who responded to cathodal stimulation in general were larger and spanned through both the frontal and temporal language areas. Because only one patient responded to right-cathodal compared to three who responded to left-cathodal stimulation, we were unable to perform subtraction analysis to depict specific areas that may be relevant to response to one cathodal montage vs. the other. However, from lesion overlap it appeared that left-cathodal patients exhibited lesions extending more superiorly and medially into the parietal and frontotemporal areas.

Another important variable that is often discounted is the extent of white matter tract damage, which measures of lesion size and location do not fully account for, but which may significantly impact response to tDCS. A large body of evidence suggests the critical role of white matter damage, especially along the arcuate fasciculus, in both the language deficits and course of recovery in PWA (Naeser et al., 1989; Marchina et al., 2011; Wang et al., 2013; Basilakos et al., 2014; Nunnari et al., 2014). The emergence of diffusion tensor imaging techniques has greatly simplified computation of the volume of damage within white matter tracts, or lesion load, which can be used to predict response to tDCS montages. In recent studies, lesion load of the arcuate fasciculus was shown to selectively predict efficiency of speech and naming ability in PWA (Marchina et al., 2011; Wang et al., 2013). Although we are unable to compute lesion load in our subjects, in future applications of this study design, pairing tDCS administration with diffusion tensor imaging, and also functional neuroimaging and/or electrophysiological measures will enable more comprehensive assessment of the relationship between changes at the neural level in response to different tDCS montages, as well as involvement of white matter damage in ensuing language outcomes. Moving forward, such multi-modal approaches may also provide some insight into the neural and structural bases of our unexpected finding related to patients' response to left-cathodal stimulation.

Intriguingly, in Phase 1, three subjects did not respond to any montages. While it is unclear why this difference exists, a general explanation could be that these subjects may differ categorically with respect to the mechanisms of language representation and aphasia recovery that may underlie response to tDCS. Lesion location and distribution may also be relevant (Martin et al., 2009). Overall, the relevance of lesion extension and response to tDCS warrants further study as it may have broad implications for the therapeutic use of tDCS in aphasic patients (Naeser et al., 1989).

Our main Phase 2 finding is encouraging and adds to a growing body of literature for tDCS' use in treating aphasia (see Monti et al., 2013, for a recent review). A number of prior studies have focused on the immediate effects of tDCS on language ability by targeting either inferior frontal or temporal areas in the language network (Monti et al., 2008; Hesse et al., 2011; Jung et al., 2011; Kang et al., 2011; Vines et al., 2011; You et al., 2011). Other investigators have extended these findings by reporting benefits that persist for weeks after stimulation (Baker et al., 2010; Fiori et al., 2011; Floel et al., 2011). A few investigators have reported findings related to the long-term therapeutic benefits of tDCS (Manenti et al., 2015), but these findings are mixed (Marangolo et al., 2013a; Polanowska et al., 2013). As investigators continue to explore tDCS as a potential therapy for aphasia, additional studies of long-term efficacy are needed.

This study has clear limitations. Because our study was structured to only recruit subjects who responded to at least one montage optimally into Phase 2, the sample size for the latter portion of the study was small. Also, the study design does not allow insight into whether patients who did not respond to any montage would have shown improvement if they had undergone treatment. The incomplete-crossover design, which allowed all subjects to eventually receive real-tDCS, gave rise to unequal subject groups and interfered with direct comparison of the real and sham conditions.

In the absence of patient-specific current-modeling, the localization of current flow during tDCS can be unpredictable in patients with brain lesions (Datta et al., 2011). This makes it difficult to comment on the exact areas that may have received stimulation in our study, particularly in patients who received left-hemispheric tDCS. Our own prior modeling in normals using a similar montage suggests that regions of highest current density are confined to the hemisphere of the active electrode (Chrysikou et al., 2013), however this remains to be verified in patients with strokes (except see Datta et al., 2011).

Of note, in this study, we placed the active electrode superior to the inferior frontal gyrus. Insofar as the left inferior frontal gyrus is often damaged in patients with non-fluent aphasia, at the study outset we had posited that this area was likely to be directly lesioned in many of the patients we would be seeking to recruit. Chronically lesioned areas of the brain are typically not comprised of viable tissue and are in fact often characterized by encephalomalacia (a CSF-filled cavity at the site of prior injury). Therefore, we decided not to place the active electrode directly over the left inferior frontal gyrus, reasoning that the direct effect on underlying structures (or perhaps more accurately the absence of brain structures) would not be clear. Secondly, since perilesional areas of the left hemisphere are known to be especially important areas for language recovery (Hamilton et al., 2011; Turkeltaub et al., 2011; Shah et al., 2013), we chose a region that was likely to be perilesional (rather than lesional) for many patients with non-fluent aphasia. The reasoning for the reverse active electrode location i.e., active electrode over a region superior to the right inferior frontal gyrus, is more complicated. We chose our right hemisphere stimulation site to be the anatomic mirror of the left hemisphere site. However, because the right inferior frontal gyrus was intact in our patients and potentially important to the recovery of reorganized language systems (reviewed in Shah et al., 2013), we wanted to choose a right hemisphere target that was also likely to result in current flow through the this region. Early modeling studies of tDCS had shown that the flow of current associated with tDCS can be quite diffuse, covering not only a large area under the active electrode, but also areas between the active and reference electrodes (Miranda et al., 2006). We reasoned that the current flow between our active frontal electrode and our contralateral mastoid reference electrode was likely to pass inferiorly from the point of placement of the active electrode. Of note, this notion of current flowing inferiorly when employing a mastoid reference electrode is supported by subsequent work with current modeling in normal subjects in which we used an active frontal lobe electrode and mastoid reference (Chrysikou et al., 2013). This is relevant because prior modeling work by Datta et al. (2011 as discussed below) does not inform the likely flow of current through the intact right hemisphere when the reference electrode is placed over the left mastoid.

The appropriate choice of location for a reference electrode can also pose a challenge for tDCS studies, particularly in patients with brain lesions. Most prior studies that have involved an active electrode over a frontal site have either employed a reference electrode over the contralateral supraorbital region (above the eye; Kang et al., 2011), or less frequently on an extracephalic site like contralateral shoulder (Monti et al., 2008; Baker et al., 2010). No prior therapeutic study in stroke patients to date has used the contralateral mastoid. However, one recent study used computational modeling to compare patterns of current flow in the brain of a patient with a left hemispheric stroke (involving inferior and medial frontal areas) when an active electrode was placed over a left frontal site and three separate reference electrode locations—right shoulder, mastoid or orbitofrontal area—were employed (Datta et al., 2011). While both right mastoid and shoulder references resulted in higher electrical fields involving posterior temporal perilesional areas, right orbitofrontal (near forehead) reference resulted in higher electrical fields in anterior frontal perilesional areas and in the spared contralesional frontal lobe. Therefore, the same active electrode position with different reference positions produced different profiles of current flow, targeting diverse perilesional areas in this patient. Considering the differences in current flow implied by these models, our selection of a contralateral mastoid reference electrode site may have preferentially facilitated recruitment of perilesional cortex as well as more inferior bilateral fronto-temporal language areas. Modeling with different reference electrode locations, particularly in stroke patients, or carrying out individualized modeling will better inform future studies to more precisely target areas under the active electrode, reference electrode, and the brain areas between them.

A lack of monitoring subjective sensory effects could raise potential concerns that real stimulation may have been distinguishable from sham in our study. Evidence regarding blinding real- vs. sham-tDCS with 2 mA are mixed, with some evidence suggesting inadequacies in blinding with 2 mA (O'connell et al., 2013), while other data suggest reliable blinding at the same current strength (Gandinga et al., 2006; Russo et al., 2013). We recommend that future studies incorporate subjective sensory judgment scales to verify blinding, particularly at higher current strengths.

Finally, while the data presented here are consistent with the notion that individualized stimulation may be a beneficial approach for treating aphasic patients using tDCS, it is important to recognize one of the inferential limitations of this investigation. While our data indicate that individuals who responded to tDCS in the short-term also exhibited longer-lasting improvement in language ability after stimulation, the design of the study does not allow further insight into whether patients who did not respond to any montages optimally would have shown improvement if they had been treated with a “sub-optimal” montage for 10 days.

Despite its caveats and limitations, the results of this preliminary study have several important implications for studies of tDCS in aphasia. First, direct comparison of all montage combinations yields a consistent response with an unexpected montage, suggesting the necessity of exploring montages beyond those indicated by the interhemispheric inhibition model or by conventional conceptions of polarity-specific effects. At the same time, our evidence suggests that individuals vary in the optimal parameters for brain stimulation, and that incorporating individualized responses into future treatment trials may be important for optimizing treatment effects in patients. Additional investigations could also use a multimodal approach such as lesion-mapping, functional neuroimaging (e.g., in healthy individuals: Holland et al., 2011; Meinzer et al., 2014), or combined tDCS and TMS to further distinguish individuals who do and do not respond to tDCS, and to characterize the neural mechanisms of responsiveness to specific stimulation montages. Finally this promising preliminary investigation further underscores the need for larger randomized controlled trials to test the long-term efficacy of tDCS as a therapy for aphasia.

### Conflict of interest statement

The authors declare that the research was conducted in the absence of any commercial or financial relationships that could be construed as a potential conflict of interest.

## Supplementary material

Supplementary Figure 1 **Structural images of all patients in Phase 1 (due to a data storage error, images for P1 are not represented) including illustrations of optimal electrode positions for P2–P7; both anode (red) and cathode (blue) electrodes are represented; LA, Left anode; RC, Right cathode; LC, Left cathode**.

## References

[B2] Anglade C. Thiel A. Ansaldo A. I. (2014). The complementary role of the cerebral hemispheres in recovery from aphasia after stroke: a critical review of literature. Brain Injury 28, 138–145. 10.3109/02699052.2013.859734 24456053

[B3] Ayache S. S. Farhat W. H. Zouari H. G. Hosseini H. Mylius V. Lefaucheur J. P. (2012). Stroke rehabilitation using noninvasive cortical stimulation: motor deficit. Expert Rev. Neurother. 12, 949–972. 10.1586/ern.12.83 23002939

[B4] Baker J. M. Rorden C. Fridriksson J. (2010). Using transcranial direct-current stimulation to treat stroke patients with aphasia. Stroke 41, 1229–1236. 10.1161/STROKEAHA.109.576785 20395612 PMC2876210

[B5] Basilakos A. Fillmore P. Rorden C. Guo D. Bonilha L. Fridriksson J. (2014). Regional white matter damage predicts speech fluency in chronic post-stroke aphasia. Front. Hum. Neurosci. 8:845. 10.3389/fnhum.2014.00845 25368572 PMC4201347

[B67] Basso A. (2010). Natural” conversation: a treatment for severe aphasia. Aphasiology 24, 466–479. 10.1080/0268703080271416524558295

[B6] Batsikadze G. Moliadze V. Paulus W. Kuo M. F. Nitsche M. A. (2013). Partially non-linear stimulation intensity-dependent effects of direct current stimulation on motor cortex excitability in humans. J. Physiol. 591, 1987–2000. 10.1113/jphysiol.2012.249730 23339180 PMC3624864

[B7] Brunoni A. R. Amadera J. Berbel B. Volz M. S. Rizzerio B. G. Fregni F. (2011). A systematic review on reporting and assessment of adverse effects associated with transcranial direct current stimulation. Int. J. Neuropsychopharmacol. 14, 1133–1145. 10.1017/S1461145710001690 21320389

[B8] Chrysikou E. G. Hamilton R. H. (2011). Noninvasive brain stimulation in the treatment of aphasia: exploring interhemispheric relationships and their implications for neurorehabilitation. Restor. Neurol. Neurosci. 29, 375–394. 10.3233/RNN-2011-0610 22124035

[B9] Chrysikou E. G. Hamilton R. H. Coslett H. B. Datta A. Bikson M. Thompson-Schill S. L. (2013). Noninvasive transcranial direct current stimulation over the left prefrontal cortex facilitates cognitive flexibility in tool use. Cogn. Neurosci. 4, 81–89. 10.1080/17588928.2013.768221 23894253 PMC3719984

[B10] Cuypers K. Leenus D. J. F. Van Wijmeersch B. Thijs H. Levin O. Swinnen S. P.. (2013). Anodal tDCS increases corticospinal output and projection strength in multiple sclerosis. Neurosci. Lett. 554, 151–155. 10.1016/j.neulet.2013.09.004 24036466

[B11] Datta A. Baker J. M. Bikson M. Fridriksson J. (2011). Individualized model predicts brain current flow during transcranial direct-current stimulation treatment in responsive stroke patient. Brain Stimul. 4, 169–174. 10.1016/j.brs.2010.11.001 21777878 PMC3142347

[B1] de Aguiar V. Paolazzi C. L. Miceli G. (2015). tDCS in post-stroke aphasia: the role of stimulation parameters, behavioral treatment and patient characteristics. Cortex 63, 296–316. 10.1016/j.cortex.2014.08.015 25460496

[B12] Feng W. W. Bowden M. G. Kautz S. (2013). Review of transcranial direct current stimulation in poststroke recovery. Top. Stroke Rehabil. 20, 68–77. 10.1310/tsr2001-68 23340073

[B13] Fiori V. Coccia M. Marinelli C. V. Vecchi V. Bonifazi S. Ceravolo M. G.. (2011). Transcranial direct current stimulation improves word retrieval in healthy and nonfluent aphasic subjects. J. Cogn. Neurosci. 23, 2309–2323. 10.1162/jocn.2010.21579 20946060

[B14] Floel A. Meinzer M. Kirstein R. Nijhof S. Deppe M. Knecht S.. (2011). Short-term anomia training and electrical brain stimulation. Stroke 42, 2065–2067. 10.1161/STROKEAHA.110.609032 21636820

[B15] Gandinga P. C. Hummel F. C. Cohen L. G. (2006). Transcranial DC stimulation (tDCS): a tool for double-blind sham-controlled clinical studies in brain stimulation. Clin. Neurophysiol. 117, 845–850. 10.1016/j.clinph.2005.12.003 16427357

[B16] Goedert K. M. Boston R. C. Barrett A. M. (2013). Advancing the science of spatial neglect rehabilitation: an improved statistical approach with mixed linear modeling. Front. Hum. Neurosci. 7:211. 10.3389/fnhum.2013.00211 23730283 PMC3657689

[B17] Hamilton R. Chrysikou E. Coslett B. (2011). Mechanisms of aphasia recovery after stroke and the role of noninvasive brain stimulation. Brain Lang. 118, 40–50. 10.1016/j.bandl.2011.02.005 21459427 PMC3109088

[B18] Hartwigsen G. Saur D. Price C. J. Ulmer S. Baumgaertner A. Siebner H. R. (2013). Perturbation of the left inferior frontal gyrus triggers adaptive plasticity in the right homologous area during speech production. Proc. Natl. Acad. Sci. U.S.A. 110, 16402–16407. 10.1073/pnas.1310190110 24062469 PMC3799383

[B19] Heiss W. D. Thiel A. (2006). A proposed regional hierarchy in recovery of post-stroke aphasia. Brain Lang. 98, 118–123. 10.1016/j.bandl.2006.02.002 16564566

[B20] Hesse S. Waldner A. Mehrholz J. Tomelleri C. Pohl M. Werner C. (2011). Combined transcranial direct current stimulation and robot-assisted arm training in subacute stroke patients: an exploratory, randomized multicenter trial. Neurorehabil. Neural Repair 25, 838–846. 10.1177/1545968311413906 21825004

[B21] Holland R. Leff A. Josephs O. Glea J. Desikan M. Price C. (2011). Speech facilitation by left inferior frontal cortex stimulation. Curr. Biol. 22, 1401–1407 10.1016/j.cub.2011.07.021 PMC3315006 21820308

[B22] Jacobson L. Koslowsky M. Lavidor M. (2012). tDCS polarity effects in motor and cognitive domains: a meta-analytical review. Exp. Brain Res. 216, 1–10. 10.1007/s00221-011-2891-9 21989847

[B23] Jung I. Y. Lim J. Y. Kang E. K. Sohn H. M. Paik N. J. (2011). The factors associated with good responses to speech therapy combined with transcranial direct current stimulation in post-stroke aphasic patients. Ann. Rehabil. Med. 35, 460–469. 10.5535/arm.2011.35.4.460 22506160 PMC3309227

[B24] Kang E. K. Kim Y. K. Sohn H. M. Cohen L. G. Paik N. J. (2011). Improved picture naming in aphasia patients treated with cathodal tDCS to inhibit the right Broca's homologue area. Restor. Neurol. Neurosci. 29, 141–152. 10.3233/RNN-2011-0587 21586821 PMC4886370

[B25] Kertesz A. (1982). Western Aphasia Battery Test Manual. San Antonio, TX: The Psychological Corporation.

[B26] Kessler S. K. Turkeltaub P. E. Benson J. G. Hamilton R. H. (2012). Differences in the experience of active and sham transcranial direct current stimulation. Brain Stimul. 5, 155–162. 10.1016/j.brs.2011.02.007 22037128 PMC3270148

[B27] Khedr E. M. Shawky O. A. El-Hammady D. H. Rothwell J. C. Darwish E. S. Mostafa O. M.. (2013). Effect of anodal versus cathodal transcranial direct current stimulation on stroke rehabilitation: a pilot randomized controlled trial. Neurorehabil. Neural Repair 27, 592–601. 10.1177/1545968313484808 23609526

[B28] Koch G. Bonni S. Giacobbe V. Bucchi G. Basile B. Lupo F.. (2012). Theta-burst stimulation of the left hemisphere accelerates recovery of hemispatial neglect. Neurology 78, 24–30. 10.1212/WNL.0b013e31823ed08f 22170878

[B29] Maher L. M. Kendall D. Swearengin J. A. Rodriguez A. Leon S. A. Pingel K.. (2006). A pilot study of use-dependent learning in the context of Constraint Induced Language Therapy. J. Int. Neuropsychol. Soc. 12, 843–852. 10.1017/S1355617706061029 17064447

[B30] Manenti R. Petesi M. Brambilla M. Rosini S. Miozzo A. Padovani A.. (2015). Efficacy of semantic-phonological treatment combined with tDCS for verb retrieval in a patient with aphasia. Neurocase 21, 109–119. 10.1080/13554794.2013.873062 24417248

[B31] Mansur C. G. Fregni F. Boggio P. S. Riberto M. Gallucci-Neto J. Santos C. M.. (2005). A sham stimulation-controlled trial of rTMS of the unaffected hemiphere in stroke patients. Neurology 64, 1802–1804. 10.1212/01.WNL.0000161839.38079.92 15911819

[B32] Marangolo P. Fiori V. Calpagnano M. A. Campana S. Razzano C. Caltagirone C.. (2013a). tDCS over the left inferior frontal cortex improves speech production in aphasia. Front. Hum. Neurosci. 7:539. 10.3389/fnhum.2013.00539 24046740 PMC3764371

[B33] Marangolo P. Fiori V. Caltagirone C. Marini A. (2013b). How conversational therapy influences language recovery in chronic non-fluent aphasia. Neuropsychol. Rehabil. 23, 715–731. 10.1080/09602011.2013.804847 23734669

[B34] Marchina S. Zhu L. L. Norton A. Zipse L. Wan C. Y. Schlaug G. (2011). Impairment of speech production predicted by lesion load of the left arcuate fasciculus. Stroke 42, 2251–2256. 10.1161/STROKEAHA.110.606103 21719773 PMC3167233

[B35] Martin P. I. Naeser M. A. Ho M. Doron K. W. Kurland J. Kaplan J.. (2009). Overt naming fMRI pre- and post-TMS: Two nonfluent aphasia patients, with and without improved naming post-TMS. Brain Lang. 111, 20–35. 10.1016/j.bandl.2009.07.007 19695692 PMC2803355

[B36] Medina J. Norise C. Faseyitan O. Coslett H. B. Turkeltaub P. E. Hamilton R. H. (2012). Finding the right words: Transcranial magnetic stimulation improves discourse productivity in non-fluent aphasia after stroke. Aphasiology 26, 1153–1168. 10.1080/02687038.2012.710316 23280015 PMC3532848

[B37] Meinzer M. Lindenberg R. Darkow R. Ulm L. Copland D. Floel A. (2014). Transcranial direct current stimulation and simultaneous functional magnetic resonance imaging. J. Vis. Exp. 86:e51730 Available online at: http://www.jove.com/video/51730/transcranial-direct-current-stimulation-simultaneous-functional 10.3791/51730 PMC4181345 24796646

[B38] Miranda P. C. Lomarev M. Hallett M. (2006). Modeling the current distribution during transcranial direct current stimulation. Clin. Neurophysiol. 117, 1623–1629. 10.1016/j.clinph.2006.04.009 16762592

[B39] Monti A. Cogiamanian F. Marceglia S. Ferrucci R. Mameli F. Mrakic-Sposta S.. (2008). Improved naming after transcranial direct current stimulation in aphasia. J. Neurol. Neurosurg. Psychiatry 79, 451–453. 10.1136/jnnp.2007.135277 18096677

[B40] Monti A. Ferrucci R. Fumagalli M. Mameli F. Cogiamanian F. Ardolino G.. (2013). Transcranial direct current stimulation (tDCS) and language. J. Neurol. Neurosurg. Psychiatry 84, 832–842. 10.1136/jnnp-2012-302825 23138766 PMC3717599

[B41] Moos K. Vossel S. Weidner R. Sparing R. Fink G. R. (2012). Modulation of Top-down control of visual attention by cathodal tDCS over right IPS. J. Neurosci. 32, 16360–16368. 10.1523/JNEUROSCI.6233-11.2012 23152618 PMC6794038

[B42] Mylius V. Ayache S. S. Zouari H. G. Aoun-Sebaiti M. Farhat W. H. Lefaucheur J. P. (2012). Stroke rehabilitation using noninvasive cortical stimulation: hemispatial neglect. Expert Rev. Neurother. 12, 983–991. 10.1586/ern.12.78 23002941

[B43] Naeser M. A. Martin P. I. Nicholas M. Baker E. H. Seekins H. Kobayashi M.. (2005). Improved picture naming in chronic aphasia after TMS to part of right Broca's area: An open-protocol study. Brain Lang. 93, 95–105. 10.1016/j.bandl.2004.08.004 15766771

[B44] Naeser M. A. Palumbo C. L. Helmestabrooks N. Stiassnyeder D. Albert M. L. (1989). Severe nonfluency in aphasia - role of the medial subcallosal fasciculus and other white matter pathways in recovery of spontaneous speech. Brain 112, 1–38. 10.1093/brain/112.1.1 2917272

[B45] Nitsche M. A. Paulus W. (2000). Excitability changes induced in the human motor cortex by weak transcranial direct current stimulation. J. Physiol. 527, 633–639. 10.1111/j.1469-7793.2000.t01-1-00633.x 10990547 PMC2270099

[B46] Nunnari D. Bonanno L. Bramanti P. Marino S. (2014). Diffusion tensor imaging and neurophysiologic assessment in aphasic stroke. J. Stroke Cerebrovasc. Dis. 23, 477–478 10.1016/j.jstrokecerebrovasdis.2014.07.047 25284718

[B47] O'connell N. E. Cossar J. Marston L. Wand B. M. Bunce D. De Souza L. H.. (2013). Transcranial direct current stimulation of the motor cortex in the treatment of chronic nonspecific low back pain: a randomized, double-blind exploratory study. Clin. J. Pain 29, 26–34. 10.1097/AJP.0b013e318247ec09 23221623

[B48] Oldfield R. C. (1971). The assessment and analysis of handedness: The Edinburgh inventory. Neuropsychologia 9, 97–113. 10.1016/0028-3932(71)90067-4 5146491

[B49] Pellicciari M. C. Brignani D. Miniussi C. (2013). Excitability modulation of the motor system induced by transcranial direct current stimulation: a multimodal approach. Neuroimage 83, 569–580. 10.1016/j.neuroimage.2013.06.076 23845429

[B50] Polanowska K. E. Lesniak M. M. Seniow J. B. Czepiel W. Czlonkowska A. (2013). Anodal transcranial direct current stimulation in early rehabilitation of patients with post-stroke non-fluent aphasia: a randomized, double-blind, sham-controlled pilot study. Restor. Neurol. Neurosci. 31, 761–771. 10.3233/RNN-130333 24047756

[B51] Pulvermuller F. Neininger B. Elbert T. Mohr B. Rockstroh B. Koebbel P.. (2001). Constraint-induced therapy of chronic aphasia after stroke. Stroke 32, 1621–1626. 10.1161/01.STR.32.7.1621 11441210

[B52] R Development Core Team (2013). R: A Language and Environment for Statistical Computing. Vienna: R Foundation for Statistical Computing.

[B53] Reis J. Schambra H. M. Cohen L. G. Buch E. R. Fritsch B. Zarahn E.. (2009). Noninvasive cortical stimulation enhances motor skill acquisition over multiple days through an effect on consolidation. Proc. Natl. Acad. Sci. U.S.A. 106, 1590–1595. 10.1073/pnas.0805413106 19164589 PMC2635787

[B54] Rosen H. J. Petersen S. E. Linenweber M. R. Snyder A. Z. White D. A. Chapman L.. (2000). Neural correlates of recovery from aphasia after damage to left inferior frontal cortex. Neurology 55, 1883–1894. 10.1212/WNL.55.12.1883 11134389

[B55] Russo R. Wallace D. Fitzgerald P. B. Cooper N. R. (2013). Perception of comfort during active and sham transcranial direct current stimulation: a double blind study. Brain Stimul. 6, 946–951. 10.1016/j.brs.2013.05.009 23835166

[B56] Schlaug G. Marchina S. Wan C. Y. (2011). The Use of non-invasive brain stimulation techniques to facilitate recovery from post-stroke aphasia. Neuropsychol. Rev. 21, 288–301. 10.1007/s11065-011-9181-y 21842404 PMC3176334

[B57] Shah P. P. Szaflarski J. Allendorfer J. Hamilton R. H. (2013). Induction of neuroplasticity and recovery in post-stroke aphasia by non-invasive brain stimulation. Front. Hum. Neurosci. 7:888. 10.3389/fnhum.2013.00888 24399952 PMC3870921

[B58] Sunwoo H. Kim Y. H. Chang W. H. Noh S. Kim E. J. Ko M. H. (2013). Effects of dual transcranial direct current stimulation on post-stroke unilateral visuospatial neglect. Neurosci. Lett. 554, 94–98. 10.1016/j.neulet.2013.08.064 24021804

[B59] Szekely A. Jacobsen T. D'amico S. Devescovi A. Andonova E. Herron D.. (2004). A new on-line resource for psycholinguistic studies. J. Mem. Lang. 51, 247–250. 10.1016/j.jml.2004.03.002 23002322 PMC3446821

[B60] Torres J. Drebing D. Hamilton R. (2013). TMS and tDCS in post-stroke aphasia: Integrating novel treatment approaches with mechanisms of plasticity. Restor. Neurol. Neurosci. 31, 501–515. 10.3233/RNN-130314 23719561

[B61] Turkeltaub P. E. Coslett H. B. Thomas A. L. Faseyitan O. Benson J. Norise C.. (2012). The right hemisphere is not unitary in its role in aphasia recovery. Cortex 48, 1179–1186. 10.1016/j.cortex.2011.06.010 21794852 PMC3221765

[B62] Turkeltaub P. E. Messing S. Norise C. Hamilton R. H. (2011). Are networks for residual language function and recovery consistent across aphasic patients? Neurology 76, 1726–1734. 10.1212/WNL.0b013e31821a44c1 21576689 PMC3100133

[B63] Vines B. W. Norton A. C. Schlaug G. (2011). Non-invasive brain stimulation enhances the effects of melodic intonation therapy. Front. Psychol. 2:230. 10.3389/fpsyg.2011.00230 21980313 PMC3180169

[B64] Volpato C. Cavinato M. Piccione F. Garzon M. Meneghello F. Birbaumer N. (2013). Transcranial direct current stimulation (tDCS) of Broca's area in chronic aphasia: a controlled outcome study. Behav. Brain Res. 247, 211–216. 10.1016/j.bbr.2013.03.029 23538068

[B65] Wang J. Marchina S. Norton A. Wan C. Schlaug G. (2013). Predicting speech fluency and naming abilities in aphasic patients. Front. Hum. Neurosci. 7:831 10.3389/fnhum.2013.00831 PMC3857577 24339811

[B66] You D. S. Kim D. Y. Chun M. H. Jung S. E. Park S. J. (2011). Cathodal transcranial direct current stimulation of the right Wernicke's area improves comprehension in subacute stroke patients. Brain Lang. 119, 1–5. 10.1016/j.bandl.2011.05.002 21641021

