# Supplementary material for: Individualized treatment with transcranial direct current stimulation in patients with chronic non-fluent aphasia due to stroke
Source: Front Hum Neurosci. 2015 Apr 21;9:201. doi: 10.3389/fnhum.2015.00201 (PMC4404833; doi:10.3389/fnhum.2015.00201)
Supplement: Supplementary file 1 [file Table1.DOCX]

**Supplementary Table 1.** Descriptive statistics and statistical output comparing 10 lists consisting of 80 pictures each, extracted from the IPNP database, used in Phase 1 as the pre- and post-stimulation testing stimuli. Note that some items overlap across lists because of the limited size of the IPNP database.

|  | Word frequency (CELEX) | Word length in syllables |
| --- | --- | --- |
| List 1 | 2.75 ± 1.32 | 1.64 ± 0.68 |
| List 2 | 2.79 ± 1.31 | 1.62 ± 0.73 |
| List 3 | 2.83 ± 1.35 | 1.62 ± 0.68 |
| List 4 | 2.84 ± 1.37 | 1.64 ± 0.67 |
| List 5 | 2.73 ± 1.40 | 1.61 ± 0.68 |
| List 6 | 2.81 ± 1.44 | 1.62 ± 0.68 |
| List 7 | 2.76 ± 1.36 | 1.65 ± 0.71 |
| List 8 | 2.77 ± 1.48 | 1.64 ± 0.71 |
| List 9 | 2.79 ± 1.41 | 1.64 ± 0.75 |
| List 10 | 2.77 ± 1.41 | 1.64 ± 0.68 |
|  | **F(9, 790) = 0.05, p>0.05)** | **F(9, 790) = 0.02, p>0.05)** |
