# Supplementary material for: Individualized treatment with transcranial direct current stimulation in patients with chronic non-fluent aphasia due to stroke
Source: Front Hum Neurosci. 2015 Apr 21;9:201. doi: 10.3389/fnhum.2015.00201 (PMC4404833; doi:10.3389/fnhum.2015.00201)
Supplement: Supplementary file 2 [file Table2.DOCX]

| **Supplementary Table 2.** Pre- and post-stimulation and corresponding change (Δ) in naming accuracy (post versus pre) in Phase 1 | | | | | | | | | | | | | | | | | | |
| --- | --- | --- | --- | --- | --- | --- | --- | --- | --- | --- | --- | --- | --- | --- | --- | --- | --- | --- |
|  | **Left Anode** | | | **Right Anode** | | | **Left Cathode** | | | **Right Cathode** | | | **Sham** | | | **Entered Phase 2** | **Partial crossover** | |
|  | pre | post | Δ | pre | post | Δ | pre | post | Δ | pre | post | Δ | pre | post | Δ |  |  |  |
| P1 | 8 | 10 | 2 | 4 | 2 | -2 | 4 | 6 | 2 | 9 | 7 | -2 | 5 | 5 | 0 | x | Real only |  |
| P2* | 1 | 5 | 4 | 2 | 3 | 1 | 1 | 4 | 3 | 3 | 4 | 1 | 1 | 0 | -1 | x | - | |
| P3^+^ | 0 | 1 | 1 | 1 | 1 | 0 | 1 | 2 | 1 | 0 | 4 | 4 | 1 | 2 | 1 | x | Sham | - |
| P4 | 67 | 73 | 6 | 70 | 67 | -3 | 63 | 68 | 5 | 65 | 68 | 3 | 66 | 66 | 0 | x | Sham | Real |
| P5 | 27 | 26 | -1 | 17 | 22 | 5 | 21 | 33 | 12 | 21 | 18 | -3 | 21 | 16 | -5 | x | Real only |  |
| P6 | 43 | 34 | -9 | 23 | 30 | 7 | 32 | 51 | 19 | 34 | 35 | 1 | 36 | 35 | -1 | x | Sham | Real |
| P7 | 51 | 52 | 1 | 45 | 50 | 5 | 44 | 52 | 8 | 43 | 43 | 0 | 49 | 47 | -2 | x | Real only |  |
| P8 | - | - | - | 61 | 47 | -14 | 56 | 64 | 8 | 48 | 60 | 12 | 67 | 56 | -11 |  |  |  |
| P9 | 1 | 3 | 2 | 2 | 4 | 2 | 3 | 4 | 1 | 4 | 7 | 3 | 1 | 8 | 7 |  |  |  |
| P10 | 0 | 0 | 0 | 0 | 0 | 0 | 0 | 1 | 1 | 0 | 1 | 1 | 0 | 0 | 0 |  |  |  |
| P11 | 52 | 54 | 2 | 52 | 48 | -4 | 50 | 37 | -13 | 44 | 41 | -3 | 49 | 48 | -1 |  |  |  |
| P12 | 4 | 6 | 2 | 10 | 7 | -3 | 10 | 4 | -6 | 9 | 5 | -4 | 10 | 8 | -2 |  |  |  |

*P2 declined participation after Phase 1;

^+^P3 completed only the sham arm of Phase 2
