# Supplementary material for: Individualized treatment with transcranial direct current stimulation in patients with chronic non-fluent aphasia due to stroke
Source: Front Hum Neurosci. 2015 Apr 21;9:201. doi: 10.3389/fnhum.2015.00201 (PMC4404833; doi:10.3389/fnhum.2015.00201)
Supplement: Supplementary file 3 [file Table3.DOCX]

**Supplementary Table 3.** Baseline (B) WAB AQ and subset scores in subjects who entered Phase 2.

|  |  | **Baselines** | | |
| --- | --- | --- | --- | --- |
|  | WAB | B1 | B2 | B3 |
| **P1** | AQ | 29.1 | 26.7 | 25.5 |
|  | SS | 5 | 3 | 2 |
|  | AVC | 6.25 | 6.95 | 6.95 |
|  | Rep | 1.6 | 1.2 | 1.4 |
|  | Nam | 1.7 | 2.2 | 2.4 |
| **P3** | AQ | 26.1 | 30.2 | 29.2 |
|  | SS | 3 | 5 | 4 |
|  | AVC | 5.85 | 6.7 | 6.6 |
|  | Rep | 3.1 | 1.8 | 2.5 |
|  | Nam | 1.1 | 1.6 | 1.5 |
| **P4** | AQ | 84.9 | 83.2 | 83.6 |
|  | SS | 14 | 14 | 15 |
|  | AVC | 10 | 10 | 10 |
|  | Rep | 7.9 | 8.2 | 7.6 |
|  | Nam | 9.3 | 9.4 | 9.2 |
| **P5** | AQ | 48.1 | 49.2 | 48.8 |
|  | SS | 10 | 10 | 10 |
|  | AVC | 6.95 | 7.3 | 7 |
|  | Rep | 4.3 | 4.2 | 4 |
|  | Nam | 2.8 | 3.1 | 3.4 |
| **P6** | AQ | 65.3 | 69.3 | 70.0 |
|  | SS | 12 | 13 | 13 |
|  | AVC | 7.45 | 8.35 | 7.9 |
|  | Rep | 6 | 5.8 | 6.2 |
|  | Nam | 7.2 | 7.5 | 7.9 |
| **P7** | AQ | 82.8 | 84.3 | 84 |
|  | SS | 18 | 18 | 18 |
|  | AVC | 8.7 | 8.15 | 8.8 |
|  | Rep | 6.9 | 7.7 | 7.2 |
|  | Nam | 7.8 | 8.3 | 8 |
