# Supplementary figures and images for: Individualized treatment with transcranial direct current stimulation in patients with chronic non-fluent aphasia due to stroke
Source: Front Hum Neurosci. 2015 Apr 21;9:201. doi: 10.3389/fnhum.2015.00201 (PMC4404833; doi:10.3389/fnhum.2015.00201)

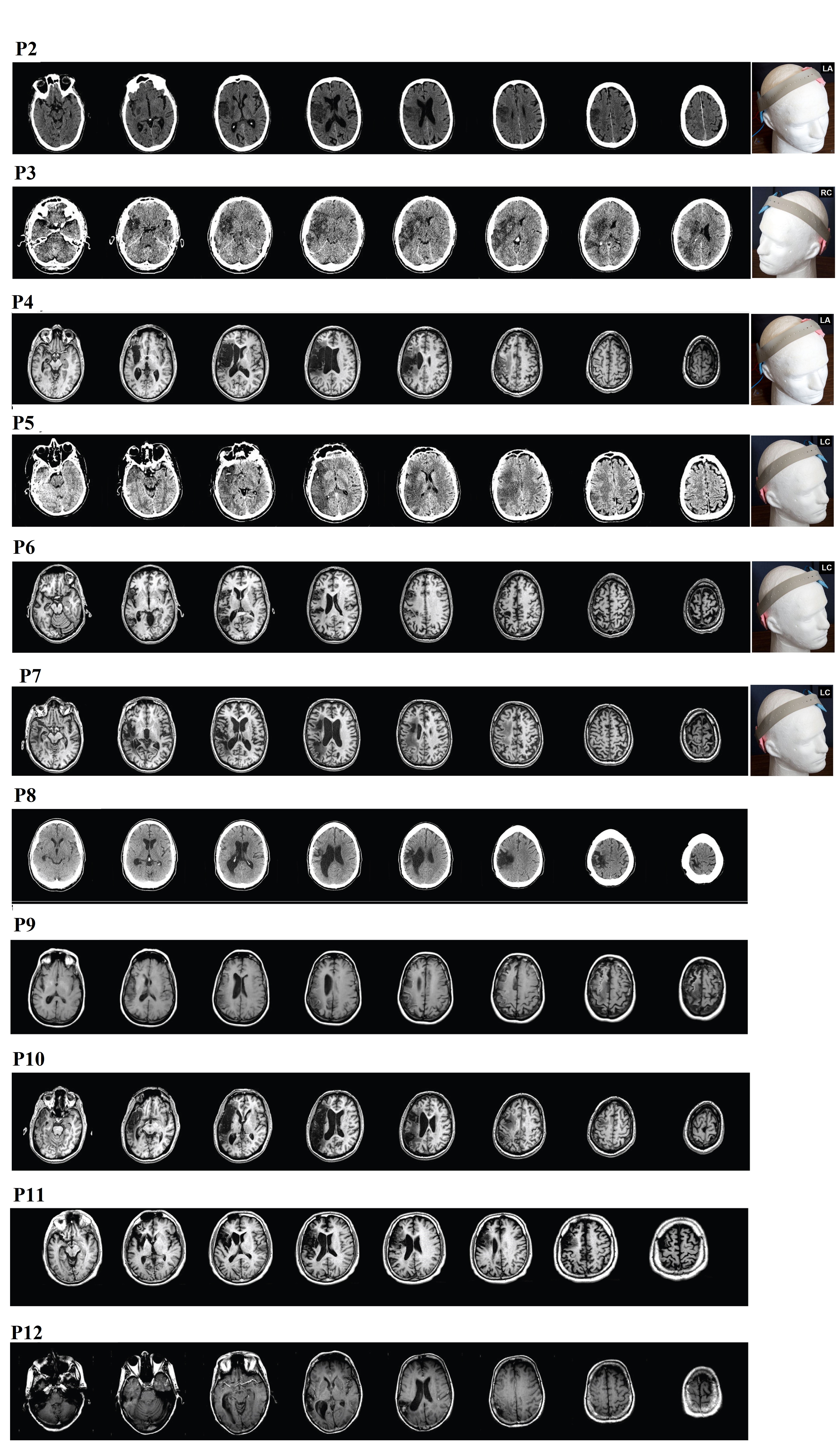

Supplement: Supplementary Figure 1 — Structural images of all patients in Phase 1 (due to a data storage error, images for P1 are not represented) including illustrations of optimal electrode positions for P2–P7; both anode (red) and cathode (blue) electrodes are represented; LA, Left anode; RC, Right cathode; LC, Left cathode. [file Image1.JPEG]
